# A Single Nucleotide in Stem Loop II of 5′-Untranslated Region Contributes to Virulence of Enterovirus 71 in Mice

**DOI:** 10.1371/journal.pone.0027082

**Published:** 2011-11-01

**Authors:** Ming-Te Yeh, Shainn-Wei Wang, Chun-Keung Yu, Kuei-Hsiang Lin, Huan-Yao Lei, Ih-Jen Su, Jen-Ren Wang

**Affiliations:** 1 The Institute of Basic Medical Sciences, National Cheng Kung University, Tainan, Taiwan; 2 Center of Infectious Disease and Signaling Research, National Cheng Kung University, Tainan, Taiwan; 3 Institute of Molecular Medicine, National Cheng Kung University, Tainan, Taiwan; 4 Department of Microbiology and Immunology, National Cheng Kung University, Tainan, Taiwan; 5 Department of Laboratory Medicine, Kaohsiung Medical University, Kaohsiung, Taiwan; 6 National Institute of Infectious Diseases and Vaccinology, National Health Research Institutes, Tainan, Taiwan; 7 Department of Medical Laboratory Science and Biotechnology, National Cheng Kung University, Tainan, Taiwan; University of Cambridge, United Kingdom

## Abstract

**Background:**

Enterovirus 71 (EV71) has emerged as a neuroinvasive virus responsible for several large outbreaks in the Asia-Pacific region while virulence determinant remains unexplored.

**Principal Findings:**

In this report, we investigated increased virulence of unadapted EV71 clinical isolate 237 as compared with isolate 4643 in mice. A fragment 12 nucleotides in length in stem loop (SL) II of 237 5′-untranslated region (UTR) visibly reduced survival time and rate in mice was identified by constructing a series of infectious clones harboring chimeric 5′-UTR. In cells transfected with bicistronic plasmids, and replicon RNAs, the 12-nt fragment of isolate 237 enhanced translational activities and accelerated replication of subgenomic EV71. Finally, single nucleotide change from cytosine to uridine at base 158 in this short fragment of 5′-UTR was proven to reduce viral translation and EV71 virulence in mice. Results collectively indicated a pivotal role of novel virulence determinant C158 on virus translation *in vitro* and EV71 virulence *in vivo*.

**Conclusions:**

These results presented the first reported virulence determinant in EV71 5′-UTR and first position discovered from unadapted isolates.

## Introduction

Enterovirus 71 (EV71), a member of the genus *Enterovirus* of *Picornaviridae* family, is a non-enveloped virus with positive, single-stranded RNA of about 7400 nt [1]. Its viral genome encodes a large polyprotein with a single open reading frame (ORF) flanked by 5′-untranslated region (UTR) and 3′-UTR. Polyprotein divides into three regions [2]: P1 containing capsid proteins VP1, VP2, VP3 and VP4; P2 and P3 containing non-structural proteins (2A, 2B, 2C, 3A, 3B, 3C and 3D) crucial to virus replication. Genome translation is initiated by a cap-independent mechanism mediated by internal ribosome entry site (IRES) in highly structured 5′-UTR [3], [4]. Six major stem loop (SL) structures essential to viral RNA replication [5] and translation [6], [7] were identified within picornavirus 5′-UTR [2], [8].

Clinical EV71 manifestations include hand-foot-and-mouth disease (HFMD), herpangina and severe neurological complications like aseptic meningitis, brainstem encephalitis, and acute flaccid paralysis in children under five years of age [9]. Neurovirulence of EV71 first drew public concern in 1975 in Bulgaria when 44 people died of polio-like disease [10]. EV71 was correlated with epidemics of neuroinvasive diseases in New York, Malaysia, Japan, Australia, Taiwan, China, Hungary and Hong Kong [11], [12], [13], [14], [15], [16], [17], [18]; it is thus regarded as an emerging neurotropic enterovirus besides poliovirus.

Molecular determinant for EV71 virulence remains unexplored [19], [20]. With generation of mouse-adapted EV71, glycine (G) to glutamic acid (E) change at amino acid 145 in VP1 was identified as determinant for the adapted virulence [21], [22]. Adaptation-derived mutation from lysine (K) to isoleucine (I) at amino acid 149 of VP2 improves virus replication in CHO cells [21], [22]. Mutations in capsid proteins as receptor binding sites determining host range were identified in these adapted strains, most proven responsible for adapted virulence. This raises a possibility of other virulence determinants' effects masked by species tropism.

Virulence determinant of other enteroviruses has been reported. Nucleotide 480, 481 and 472 in poliovirus 5′-UTR were cited as neurovirulence determinants of poliovirus Type 1, 2 and 3, respectively [23], [24], [25]. A single adenine (A) to guanine (G) change at nucleotide 103 in 5′-UTR attenuates poliovirus neurovirulence [26]. Also, SL II in 5′-UTRs of coxsackievirus (CV) B1 and B3 determines their cardiovirulence phenotype [27], [28], [29]. These studies reported contributions of 5′-UTR to viral virulence, while few reports currently indicate the importance of 5′-UTR to EV71 virulence.

To determine whether the 5′-UTR of naturally circulating EV71 contributes to virulence as other enteroviruses, we correlated 5′-UTR and differential virulence of clinical isolates in mice. We further mapped virulence determinant using recombinant viruses generated from a series of infectious clones harboring chimeric 5′-UTRs. Effect of the identified determinant on virus translation and replication *in vitro* was examined via bicistronic plasmids and subgenomic replicons. Tissue viral load was also examined to gauge effect of the determinant on virus replication *in vivo*.

## Materials and Methods

### Ethics Statement

All experiments were conducted in accordance with the guidelines of Laboratory Animal Center of National Cheng Kung University. The Institutional Animal Care and Use Committee of National Cheng Kung University approved all animal protocols. (Approved protocol No. IACUC-097026).

### Cells and Viruses

RD cells (ATCC CCL-136™) were cultured in DMEM medium supplemented with 10% fetal bovine serum (FBS), L929 cells (ATCC CCL-1™) were cultured in EMEM medium with 10% FBS and 1% sodium pyruvate added. RD cells were used for infectious RNA transfection, virus production and plaque assay. L929 cells were used for IRES activity assay. Isolate 4643 was isolated in 1998 in Virology Laboratory of National Cheng Kung University Hospital, isolate 237 obtained in 1986 at Kaohsiung Medical University Hospital. Mouse-adapted MP4 generated by four serial passages in brains of mice was used as backbone for gene manipulations to study EV71 virulence in mice [30].

#### Plasmid constructions

Standard molecular cloning techniques were used in all constructions [31]. All primer sequences are provided in supporting information (Table S1 for full-length infectious clones and chimeric 5′-UTRs, Table S2 for point mutations, and Table S3 for bicistronic plasmid and replicons).

#### Full-length EV71 infectious DNA clones

Viral genomic RNAs of MP4, 4643 and 237 were extracted using Viral Nucleic Acid Extraction Kit (Geneaid). RNAs were reverse-transcribed with primer RT-50 and SuperScript II reverse transcriptase (Invitrogen). The cDNAs were PCR-amplified with primers 4643F and RT-50 by using KOD plus DNA polymerase (Toyobo). The 7.4-kb genome of MP4, 4643 and 237 were digested with *EcoRI* and *EagI* (New England Biolabs) and then ligated to a pre-digested modified pUC19.

#### Infectious clones harboring various 5′-UTRs

The infectious clone 4643M was constructed by replacing 5′-UTR of template MP4 with sequence of 4643. Briefly, 4643 5′-UTR was PCR-amplified from full-length 4643 infectious clone with primers 4643F and R-EV71-5UTR. Partial VP4 gene of MP4 was amplified with primers MP4-VP4N and R-MP4-*BbvCI*. Two PCR products were mixed for overlapping PCR using primers 4643F and R-MP4-*BbvCI*. Purified PCR products were inserted into template MP4 between *EcoRI* and *BbvCI* sites. Infectious 237M and other infectious clones harboring chimeric or point-mutated 5′-UTRs were likewise generated.

#### EV71 subgenomic replicons

Subgenomic replicon of isolate 4643 was constructed as described [32]. Chimeric or point-mutated 5′-UTRs were PCR-amplified with primers 4643F and 5-UTR-*XmaI* from infectious clones described above and inserted to 4643 replicon between *EcoRI* and *XmaI* sites.

#### Bicistronic plasmids

Bicistronic plasmids contain beta-galactosidase gene followed by EV71 5′-UTR and firefly luciferase gene. DNA fragments from 5′-UTR to firefly luciferase gene were PCR-amplified with primers 4643F and R-*XhoI*-FLuc from various replicons constructed as described above. The fragment was inserted to pCMV vector (Stratagene) with *EcoRI* and *XhoI* sites. The β-galactosidase gene was then inserted to *NotI* and *BamHI* site.

#### Transcription *in vitro*

Replicon and infectious RNAs were transcribed *in vitro* from *EagI*-linearized DNA pladmids using MEGAscript Kit (Ambion). RNAs were purified with Sephadex G-50 spin columns (GE Healthcare) and quantified with Qubit RNA BR Assay Kit (Invitrogen). RNA integrity was tested by standard agarose gel electrophoresis.

#### Virus production from infectious DNA clones

Infectious RNAs were transfected into RD cells with TransMessenger Transfection Reagent (Qiagen) following manufacturer's instruction. Transfected cells were incubated at 37°C and harvested until 90% of cells developed cytopathic effect (CPE). Titers of experimental recombinants were determined by plaque assay.

#### Transfection and reporter activity assay

Bicistronic DNA plasmids were transfected to L929 cells with TurboFect *in vitro* Transfection Reagent (Fermentas) according to manufacturer's protocol. PBS washed cells were lysed in 1X Reporter Lysis Buffer (Promega) upon harvest. Reporter activities were analyzed with Luciferase Assay System (Promega) and β-Galactosidase Assay System (Promega). For virus replication assay, replicon RNAs were transfected to L929 cells with TransMessenger Transfection Reagent (Qiagen); cell lysates were harvested at intervals with 1X Reporter Lysis Buffer, as described earlier. All reporter activity assays were performed in triplicate and presented as relative fold change.

#### Sequences and RNA secondary structure prediction

Full-length genomic sequences of EV71 clinical isolates 237 (FJ357380) and 4643 (AF304458) are available on Genebank. RNA secondary structures were predicted by mfold [33] and RNAStructure Version 5.3 [34] with pre-set parameters; drawings were produced with RnaViz [35].

#### Western blot

Cell lysates were separated on 10% SDS-polyacrylamide gels and transferred to polyvinylidene difluoride membranes. EV71 proteins were probed with MAb979 (Millipore) in 1% BSA at 1∶2000 dilution. HRP-conjugated anti-mouse IgG (Abcam) diluted in 1% BSA at 1∶5000 dilutions was used to probe MAb979. Protein signals were developed in ECL reagents (Perkin Elmer) and exposed to X-ray films (Kodak). Antibody against β-actin (Sigma) was included as control, band density rated with ImageJ.

#### Animal experiments

Specific-pathogen-free, three-day-old Institute of Cancer Research (ICR) mice (from Laboratory Animal Center of National Cheng Kung University) were intraperitoneally (i.p.) or intracranially (i.c.) inoculated with pre-determined dose of various viruses. Control mice were given control viral medium instead of virus suspension. Mice were observed twice daily for clinical signs, body weight and survival for 14 days. Clinical signs were scored: 0, healthy; 1, ruffled fur and hunchbacked appearance; 2, wasting; 3, limb weakness; 4, limb paralysis; 5, moribund or dead [36]. To determine tissue viral load, mice were perfused with saline after euthanasia. Selected tissues were collected aseptically, weighted and stored at -70°C; samples homogenized in viral medium, disrupted by three freeze-thaw cycles, then centrifuged. Virus titers in clarified supernatants were determined by plaque assay and expressed as log pfu per mg of tissue.

#### Statistics

Statistical analyses were performed using GraphPad Prism 5 (GraphPad). Relative translational activity was analyzed by t-test with results plotted as mean ± standard error of the mean (SEM), survival rates by log-rank analysis and *P* value of <0.05 significant.

## Results

### 5′-UTR correlates with distinct virulence of two unadapted EV71 isolates

**Figure 1:**
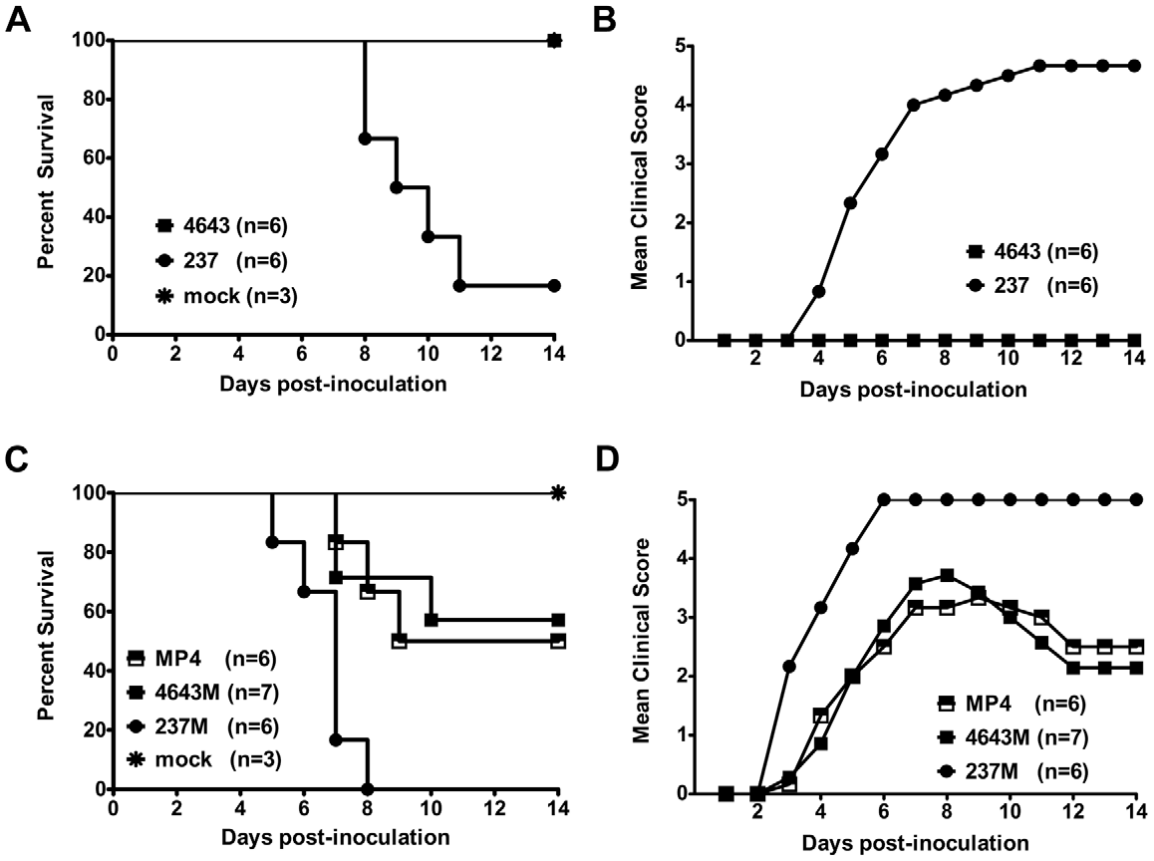
The 5′-UTR correlates with the distinct virulence of EV71 in mice. Three-day-old ICR mice were i.p. inoculated with unadapted EV71 clinical isolate 4643 or 237 at 5×10^4^ pfu/mouse (A, B), and 5′-UTR-replaced recombinant virus 4643 M or 237 M and backbone MP4 at 5×10^4^ pfu/mouse (C, D). The survivals (A, C) and clinical scores (B, D) of mice were monitored for two weeks. Mock control mice were given viral medium.

Virulence of two EV71 clinical isolates 4643 and 237, without further adaptation in cell culture or animals, were compared using a mouse infection model. Isolate 237 reduced survival time and rate of infected mice as compared with isolate 4643 (16.7% vs. 100%, *P*<0.005) (Fig. 1A). Severe clinical signs appeared in 237 infected mice while 4643 caused no clinical diseases (Fig. 1B). Results indicated EV71 clinical isolate 237 as more virulent than isolate 4643 in mice.

To identify genetic determinant for the differential virulence of EV71 clinical isolates 4643 and 237, full-length viral genomes of the two isolates were cloned and sequenced. Nucleotide sequence of 5′-UTRs and 3′-UTRs, and amino acid sequence of coding regions of the two isolates were analyzed. Phylogenetic analysis of VP1 region classified isolate 4643 as C2 genotype, and 237 as B1 (data not shown). The two isolates shared 85.1 % identity for 5′-UTR, and 88.8 % for 3′-UTR. Amino acid identity for coding regions of the two isolates was 89.9 %. These analyses revealed numerous genetic variations, located throughout the entire viral genome, that were potential molecular determinant for the differential virulence of EV71 in mice.

**Figure 2:**
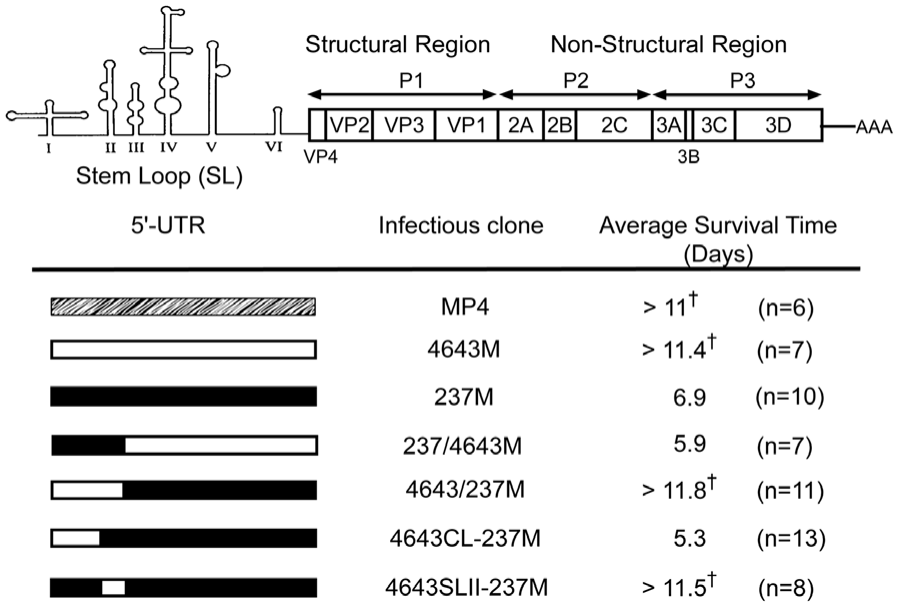
Molecular determinant of EV71 virulence in mice was mapped to SL II. Genomic structure of EV71 shown in the upper part and schematic presentation of the compositions of 5′-UTRs of infectious clones are shown in the lower panel. Open, closed, and slash box represent sequence of isolates 4643, 237, and MP4 respectively. Number and average survival time of mice infected with these clone-derived viruses were also indicated. † Survival time of mice alive on Day 14 post-inoculation was calculated as 14.

The contribution of 5′-UTR to enterovirus virulence has been extensively reported as mentioned earlier, contribution of 5′-UTR to EV71 virulence was thus first examined. To study EV71 virulence in a mouse infection model, a mouse-adapted MP4, recovered by four serial passages of isolate 4643 in brains of mice, was used as backbone for gene manipulations [30]. To determine correlation between EV71 5′-UTR and differential virulence in mice, 5′-UTR of the backbone MP4 was replaced by sequences of isolates 4643 and 237 (Fig. 2). Virulence of resulted recombinant viruses 4643 M, 237 M, and backbone MP4 were tested in mice. No 237 M infected mice survived at Day 8 post-inoculation, while over 50% of 4643 M inoculated mice survived 14 days after inoculation (0% vs. 57%, *P*<0.01) (Fig. 1C). Virus 237M caused paralysis and mortality in all cases; four of seven infected by 4643M were paralyzed, three dead (Fig. 1D). Survival rate and clinical scores of MP4-infected mice were both comparable to 4643M infection (50% vs. 57%, *P*>0.05) (Fig. 1C and 1D). Average survival time (AST) for MP4, 4643M and 237M inoculated mice were >11, >11.4, and 6.9 days (Fig. 2), respectively, evidently correlation of 5′-UTR with differential virulence of the two unadapted EV71 clinical isolates in mice.

To confirm the increased virulence of isolate 237 was not a B genotype-specific phenotype, we tested the effect of 5′-UTR from another B genotypic EV71 clinical isolate, N7008, which showed comparable virulence with isolate 4643 in mice. Mice infected with virus N7008M (MP4 backbone with N7008 5′-UTR) also showed increased survival rate as compared with 237M (60% vs. 0%, *P*<0.05) (Fig. S1). This confirmed that the increased virulence of isolate 237 in mice is specific to 5′-UTR, but not a general genotypic phenotype.

### Mapping of virulence determinant in EV71 5′-UTR

To locate virulence determinant in 5′-UTR, infectious clones harboring chimeric 5′-UTR were constructed and recombinant viruses generated were inoculated into mice (Fig. 2). AST of mice infected with 237/4643M, 4643CL-237M were 5.9 and 5.3 days, respectively, while 4643/237M and 4643SLII-237M infected mice survived more than 11.5 days (Fig. 2). Presence of 4643 SL II prolonged AST of mice, suggesting a dominant role of SL II on EV71 virulence.

**Figure 3:**
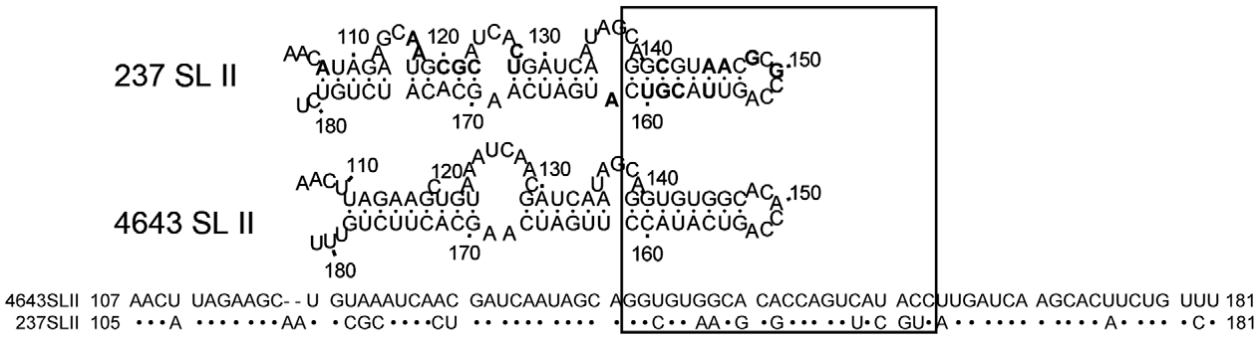
Multiple genetic variations concentrate in the SL II upper domain. Secondary structures of SL II of isolate 237 and 4643 predicted using RNA Dyalign of RNAstructure Version 5.3 are shown in the upper part. Sequence alignment of the two SL IIs revealed 20 nucleotide variations, indicated as bold in predicted secondary structure of 237 SL II, 10 concentrated from nucleotide 142 to 162 which form the SL II upper domain as boxed.

**Figure 4:**
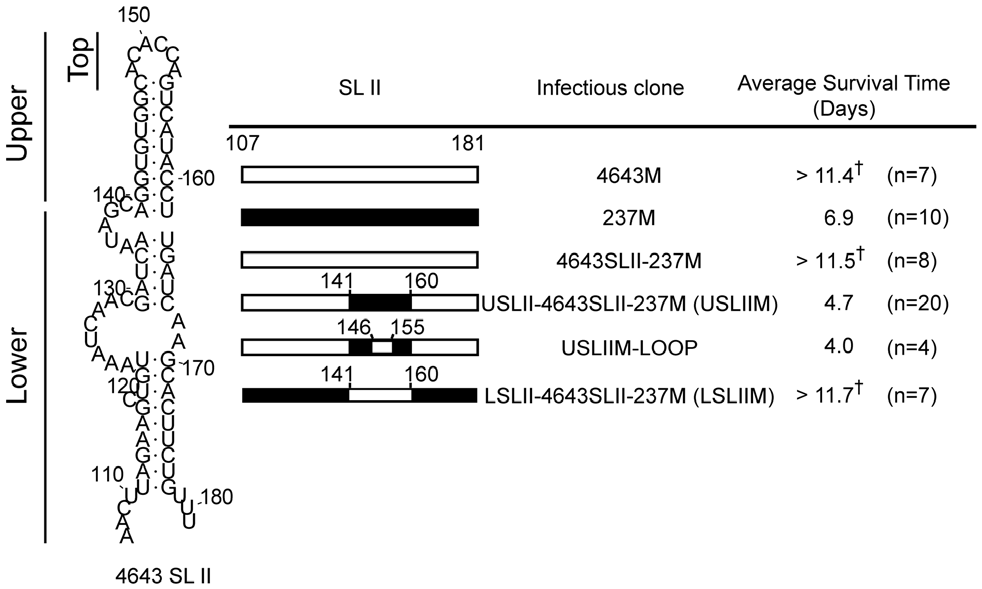
Short stem in the upper domain of SL II determines EV71 virulence in mice. The RNA secondary structure of 4643 SL II predicted, as described in Materials and Methods, is shown in the left panel. Composition of chimeric SL IIs of infectious clones is shown in the middle panel. Open and closed boxes represent sequence of isolates 4643 and 237, respectively. USLII and LSLII were generated by replacing nucleotides in the upper or lower half of 4643 SL II with corresponding sequences of isolate 237. Top loop of USLII was further replaced with corresponding sequence of isolate 4643 to generate USLII-LOOP. The average survival time of mice infected with recombinant viruses generated from these clones is shown in the right panel. Base numbers are from EV71 isolate 4643. † Survival time of mice alive on Day 14 post- inoculation was calculated as 14.

SL II in EV71 5′-UTR is secondary structure made of about 75 nucleotides in length (Fig. 3). EV71 isolates 4643 and 237 share 74% sequence identity in SL II. Among the 20 varied nucleotides, 10 located in a short region of nt. 142–162 which formed the upper domain of 4643 SL II (Fig. 3). To ferret out virulence determinant in SL II, we introduced chimeric SL II derived from isolates 4643 and 237, based on these sequence alignment and predicted secondary structure, to backbone 4643SLII-237M. Clone USLIIM contains upper SL II of 237 and lower half from 4643; LSLIIM contains upper SL II of 4643 and lower domain from 237. Top loop of USLIIM SL II was restored to sequence of 4643 to generate USLIIM-LOOP (Fig. 4). Mice infected with USLIIM and USLIIM-LOOP showed AST of 4.7 and 4 days, respectively. Upper stem of 4643 SL II prolonged AST of infected mice to >11.7 days, indicating the short fragment as attenuating virulence of USLIIM in mice (LSLIIM, Fig. 4). Collectively, results suggested a molecular determinant of EV71 virulence in mice located in the SL II upper stem, which is made of nt. 141–146 and nt. 155–160 of 5′-UTR.

### Upper stem of SL II in EV71 5′-UTR affects viral translation and replication

**Figure 5:**
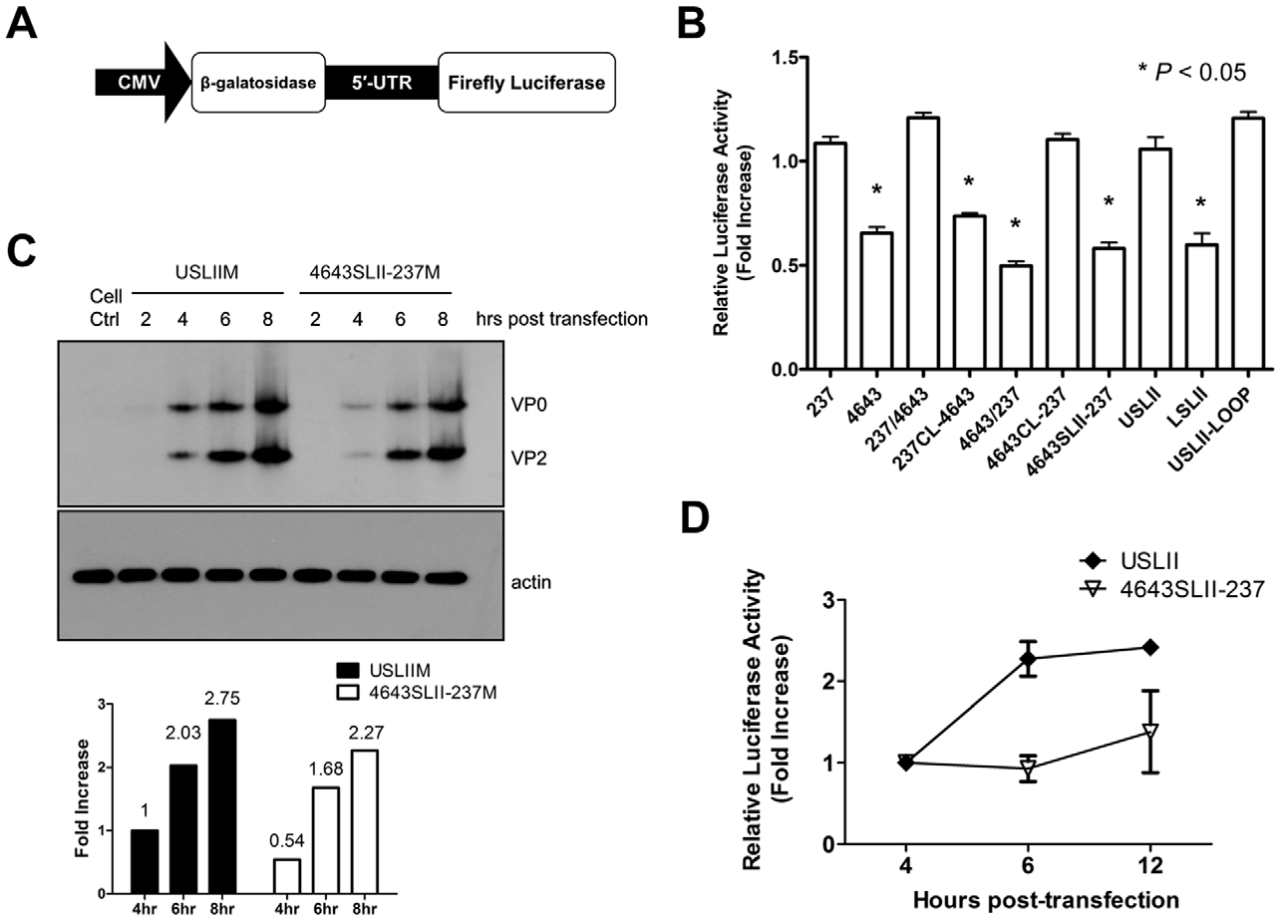
Short fragment of SL II regulated viral translation and replication *in vitro.* (A) Construction of dual reporter plasmid to determine translational activity mediated by EV71 5′-UTR. (B) Relative translational activities mediated by various 5′-UTRs. Dual reporter plasmids with various 5′-UTR were transfected into L929 cells, firefly luciferase activity determined 24 hours post-transfection and normalized with β-galactosidase activity. Relative translational activity to isolate 237 is shown as fold increase. (C) Effect of SL II on virus translational activity. Infectious RNAs USLIIM and 4643SLII-237M were transfected into L929 cells, lysates harvested at 2, 4, 6 and 8 hours post-transfection. Western blot was performed by MAb979 to detect viral translated products. Collective band density of each time-point was rated with ImageJ and expressed as fold increase relative to band density of USLIIM at four hours post-transfection. Western blot analysis of actin was included as control for normalization. (D) Effect of SL II on viral replication. Replicon RNAs of USLII and 4643SLII-237 were transfected into L929 cells and the cell lysates were harvested at 4, 6 and 12 hours post-transfection; luciferase activities at 4 hours reflect transfection efficiency. Their replication rates were expressed as fold increase of firefly luciferase activity at 6 or 12 hours versus 4 hours post-transfection.

To study effect of the SL II upper stem on viral translation, bicistronic plasmids (Fig. 5A) harboring chimeric 5′-UTRs were constructed and transfected into L929 cells; reporter activities were determined. Presence of 4643 SL II upper stem correlated with reduced translational activity as compared with 237 in L929 cells (4643, 237CL-4643, 4643/237, 4643SLII-237 and LSLII in Fig. 5B) (all *P*<0.005) while SL II upper stem of 237 enhanced translational activity (237, 237/4643, 4643CL-237, USLII and USLII-LOOP) (all *P*<0.005), indicating SL II upper stem as regulating translational activity of EV71.

Effect on viral translation was further confirmed by monitoring viral translated products in full-length viral RNA transfected cells. Western blot analysis showed that at four hours post transfection, translated viral proteins could be detected in USLIIM transfected cells while only trace protein expression was observed in cell lysate of 4643SLII-237M transfection. Higher protein levels were probed in cells transfected with USLIIM than with 4643SLII-237M at various time points (Fig. 5C). This confirmed that the upper domain of SL II affects viral translation in cells.

Although all recombinant viruses generated in this study replicated efficiently in RD cells (human rhabdomyosarcoma), the fact that NIH3T3 (mouse fibroblast), N18 (mouse neuroblastoma) and L929 cells do not efficiently support EV71 infection led to difficulties to determine effect of SL II upper domain on growth phenotype in mouse cell lines (data not shown and [37]). Alternatively, L929 cells were transfected with replicon RNAs of USLII and 4643SLII-237, and firefly luciferase activities at 4, 6 and 12 hours post-transfection were evaluated to determine regulation of SL II upper domain on virus replication. Relative luciferase activity of USLII was significantly higher than 4643SLII-237 at 6 and 12 hours post-transfection (Fig. 5D), indicating that the SL II upper domain of EV71 5′-UTR regulates both viral translation and replication.

### Upper domain of SL II affects EV71 replication

**Figure 6:**
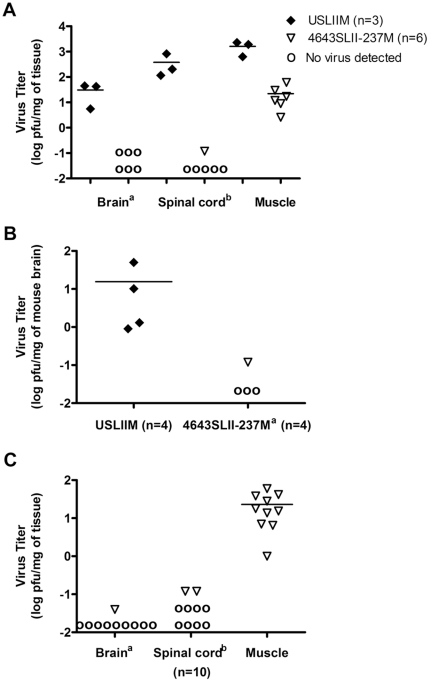
Short stem of SL II affected virus replication in mice. (A) Effect of 237 SL II upper domain on virus replication *in vivo*. Three-day-old ICR mice were i.p. inoculated with USLIIM or 4643SLII-237M at 5×10^4^ pfu/mouse. Brain, spinal cord and posterior limb muscle were recovered at Day 3 post-inoculation and virus titer determined with plaque assay. ^a^: no virus detected in brain of mice infected with 4643SLII-237M. ^b^: virus detected in only one spinal cord sample from 4643SLII-237M infected mice. (B) Effect of 237 SL II upper domain on viral replication in brain. Three-day-old ICR mice were given 2,500 pfu/dose of USLIIM or 4643SLII-237M recombinant viruses via i.c. route. Brains of infected mice were recovered post-inoculation. ^a^: virus was detected only in one brain of 4643SLII-237M infected mice. (C) Correlation between tissue viral load and paralysis in 4643SLII-237M-infected mice. Mice were i.p. inoculated with 4643SLII-237M at 5×10^4^ pfu/mouse. Brain, spinal cord and muscle were recovered after posterior limb paralysis was observed. ^a^: virus was detected in one brain of 10 paralyzed mice. ^b^: virus was detected in two spinal cord samples from 10 paralyzed mice. Results represent virus titer (log_10_ pfu) per milligram of tissue.

Effect of SL II upper domain on virus replication in mice was examined. Virus titers (log pfu/mg of tissue) in brain, spinal cord and posterior limb muscle of USLIIM infected mice were 0.9–1.6, 2.1–2.9 and 2.8–3.4, respectively. No virus was recovered from brain of mice inoculated with 4643SLII-237M, while virus titer in spinal cord was −0.92 log pfu per mg of tissue. Muscle of 4643SLII-237M infected mice was detected a reduced virus titers of 0.4–1.8 log per mg of tissue as compared with USLIIM infection (P<0.05) (Fig. 6A). Reduced replication of 4643SLII-237M in brain was confirmed by direct i.c. inoculation. Virus titers in USLIIM injected brains were about 2 logs higher than in 4643SLII-237M infected brains (*P*<0.05) (Fig. 6B), confirming that the SL II upper domain of isolate 4643 reduces virus replication in mice.

Some mice infected with 4643SLII-237M showed paralysis as with USLIIM. To determine correlation between paralysis and tissue viral load, selected tissues of 4643SLII-237M infected mice were taken for plaque assay after paralysis onset. Among 10 paralyzed mice, virus titers (log pfu/mg of tissue) of 0–1.78 were detected in muscles. Only one brain of ten was detected a low virus titer of −1.39 log pfu per mg of tissue while two spinal cord samples showed virus titers of −0.92 (Fig. 6C). This result suggested that upper SL II may also regulate neurovirulence of EV71.

### Single nucleotide substitution attenuates EV71 virulence

**Figure 7:**
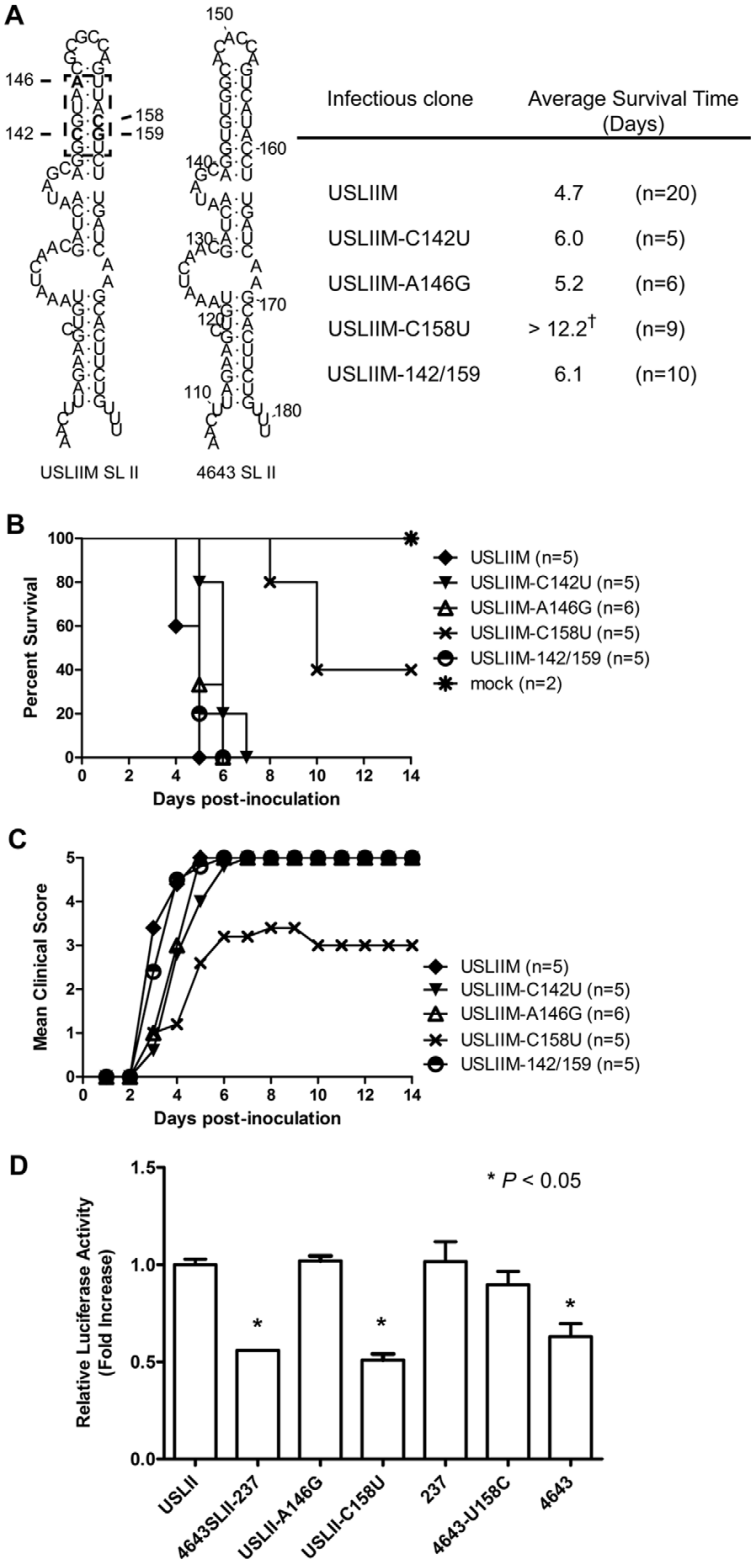
Single nucleotide substitution attenuated EV71 virulence. (A) Diagram of nucleotide substitution in SL II. Dashed rectangle represents the identified domain determining EV71 virulence. Substituted nucleotides appear in bold type; C142, A146 and C158 of USLIIM 5′-UTR were substituted with corresponding U, G and U of isolate 4643 to generate USLIIM-C142U, USLIIM-A146G and USLIIM-C158U, respectively. Clone USLIIM-142/159 contains two nucleotide substitutions of C142U and G159A. Average survival time is shown in the right panel. Recombinant viruses USLIIM, USLIIM-C142U, USLIIM-A146G, USLIIM-C158U and USLIIM-142/159 were i.p. inoculated to three-day-old ICR mice at 5×10^4^ pfu/mouse. Survival (B) and disease severity (C) were monitored for two weeks. (D) Effect of nucleotide substitution on translational activity mediated by EV71 5′-UTR. Point mutated bicistronic plasmids were transfected into L929 cells as described above, reporter activity determined 24 hours post-transfection. Relative translational activity is shown as fold increase of normalized luciferase activity to USLII.

Nucleotide substitutions were introduced into SL II upper stem of virulent USLIIM to define minimum genetic element of EV71 virulence (Fig. 7A). RNA secondary structure prediction showed that only nucleotide change of C158U caused alteration in SL II structure (Fig. S2). Viruses USLIIM, USLIIM-C142U, USLIIM-A146G and USLIIM-142/159 showed similar virulence while USLIIM-C158U prolonged AST and survival rate of infected mice as compared with USLIIM infection (40% vs. 0%, *P*<0.005) (Fig. 7A and 7B). Four of five mice infected with USLIIM-C158U showed paralysis during post-inoculation Days 6–9, with only three paralyzed mice dying at Days 8–10 (Fig. 7C). Nucleotide substitution of C158U also reduced translational activity mediated by USLII 5′-UTR (*P*<0.005), while introduction of U158C reversion to 4643 5′-UTR led to increased translational activity (P<0.05) (Fig. 7D). Results indicated that position C158 in SLII is a major determinant for viral translation and EV71 virulence.

## Discussion

EV71 Isolates 237 and 4643 were isolated in Taiwan as of 1986 and 1998, respectively. Isolate 4643 was isolated from a fatal case of encephalitis during the 1998 outbreak afflicting thousands of children, with 405 severe cases and 78 deaths. Isolate 237 was obtained from one patient during HFMD epidemic in 1986. Viral genomes of low-passage viruses were cloned as infectious types to prevent further genetic change during replication cycles, as well as for full-length genomic sequencing. No further cell culture adaptation was performed, meaning the obtained sequences were regarded as representative of original viruses. To study pathogenicity, we tested virulence of both isolates in a mouse infection model. It was interesting that, for unknown reasons, unadapted Isolate 4643 caused severe disease in humans but was avirulent to newborn mice; Isolate 237 caused only mild human infection but proved highly lethal to mice. To learn more about EV71 virulence and pinpoint the mechanism for the differential virulence, we performed this study in mice. Accumulated reports have stressed contributions of 5′-UTR in viral replication and virulence of enteroviruses [25], [26], [27], [29], [38], [39], [40], [41], [42], [43] whereas evidence on the importance of 5′-UTR to EV71 virulence is limited. Our chief objective of this study is to identify potential virulence determinant in the 5′-UTR of unadapted EV71 in mice. With generation of a series of infectious clones harboring various 5′-UTR, we mapped a genetic determinant of EV71 virulence to SL II upper stem. Further characterization of this fragment proved a single nucleotide change of C158U as sufficient to attenuate EV71 virulence. The identified determinant was proven to regulate virus translation in L929 cells and replication in mice.

The importance of SL II to viral life cycle and virulence is supported by other studies. Point mutations in SL II of Mahoney strain of poliovirus Type 1 reduce IRES-dependent viral translation and RNA replication in mouse cells, while SL II deletion mutants with 6–10 bases deleted cause non-viable phenotype [39], [44]. Nucleotide substitution at base 133 of A by G in SL II of Mahoney virus enhances efficiency of viral plus-strand RNA synthesis in Poliovirus receptor (PVR) expressing mouse cells [45]. SL II also determines cardiovirulence of CVB3 clinical isolates in a murine model [27], [46], [47]. These studies strongly support our finding that a single nucleotide determining viral virulence is located in the SL II of EV71 5′-UTR.

Virus replication is a key factor determining virulence [48], [49]. Interactions between a cloverleaf directing viral RNA replication and the IRES domain mediating viral translation greatly affect virus replication. Given the nature of positive sense of enterovirus RNA, viral genome serves as mRNA for viral protein synthesis once released into infected cells. Binding of host translational factors to the IRES domain initiates viral RNA translation. Amongst those host proteins involved, poly(rC) binding protein (PCBP) binds to the cloverleaf, and to the SL IV in IRES domain with higher affinity in the absence of viral proteins. When enough viral proteins accumulate, viral 3CD binds to the cloverleaf, yielding a 2-log increased binding affinity of PCBP to this structure, and ultimately switch from viral translation to RNA synthesis [50], [51]. Increased translational activity mediated by viral 5′-UTR hence implies faster virus replication and illustrates viral translation as an important early event regulating virus replication and virulence. Correlation between translational activity mediated by viral 5′-UTR and virulence is widely reported. Nucleotide 480, 481 and 472 which attenuates neurovirulence of poliovirus Type 1, 2 and 3, reduces viral translation in neuronal cells [23], [24], [38]. A single nucleotide mutation at position 103 attenuates neurovirulence and reduces *in vitro* translation in human neuronal cell lysate [26]. Mechanism for the regulation generally accepted: these single nucleotide changes alter RNA secondary structure and binding affinity to host factors, thereby reducing viral translation and virulence [52], [53]. Correlation between viral translation and EV71 virulence was also established in this study. The nucleotide 158 we identified as genetic determinant for EV71 virulence also regulates viral translation in L929 cells. Combined with software prediction that C158U caused alteration in RNA secondary structure of SL II, we therefore propose that nucleotide change of C158U may apply the same mechanism to determine viral translation and virulence.

Another line of evidence portends existence of tissue-specific proteins and their differential binding affinities to 5′-UTR of virulent and attenuated strains. It was reported that tissue-specific expression and differential RNA-binding abilities of polypyrimidine-tract-binding protein (PTB) and its neural-specific homologue nPTB determine cell-specific translation and neurovirulence of poliovirus [54]. In this study, differential replication abilities of virulent USLIIM and attenuated 4643SLII-237M in CNS and muscle are noticed; replication of attenuated virus in brain is not improved by direct i.c. inoculation. Since USLIIM and 4643SLII-237M share exactly the same coding region from template MP4; possibilities of differential distribution of cellular receptor that mediates viral entry or differential binding abilities to cellular receptor are excluded. The fact that viral genomes of USLIIM and 4643SLII-237M differ only in the upper SL II, part of IRES that mediates virus translation, supports a hypothesis that neural-specific protein(s) binds to SL II of USLIIM with higher affinity than 4643SLII-237M.

Besides differential virus replication in brain, recombinant virus USLIIM and 4643SLII-237M showed distinct neurovirulence in mice. Some but not all inoculated with attenuated virus 4643M, 4643SLII-237M or USLIIM-C158U displayed posterior limb paralysis at about Day 6 post-infection and gradually recovered asymptomatic status. Possible causes of paralysis are brain invasion, spinal cord injury and muscle cell destruction. Our tissue viral load analysis on paralyzed mice caused by 4643SLII-237M revealed quite a low virus titer in only one brain (−1.39 log pfu/mg), slightly higher titers in two spinal cord samples (−0.92 log pfu/mg), and a higher amount of virus in muscle (0–1.78 log pfu/mg) which we believe to cause the observed temporal paralysis. Difference of virus titer in brain and spinal cord is consistent with previous reported observation that EV71 spreads from the lower to upper segment of spinal cord, then ascends to the brain in mice [36], but it is highly unlikely that the extremely low amount of virus in brain and spinal cord causes paralysis, not to mention that only in one brain and two spinal cord samples from ten paralyzed mice were detected virus. In addition, reduced EV71 replication in mice caused by 4643 SL II, especially in muscle (∼2 Δlogs), may also limit the spread of virus to central nervous system (CNS) via retrograde axonal transport [36]. Based on the temporal paralysis phenotype and differential virus titers between CNS and muscle, we conclude that the upper SL II also contributes to neurovirulence.

Interaction between host factors and viral 5′-UTR is critical to enterovirus life cycle. Importance of host factors like IGF-II mRNA binding protein 1 (IMP-1), lupus autoantigen (La), PTB, PCBP-1, PCBP-2, heterogeneous nuclear ribonucleoprotein A1 (hnRNP A1) and hnRNP K to RNA virus replication has been extensively discussed [2], [4]. Among them, hnRNP A1 reportedly binds to SL II and SL V of EV71 5′-UTR and contributes to IRES-mediated viral translation and replication [55]. We have excluded the direct contribution of hnRNP A1 to differential virulence of EV71 according to similar response of translation activity mediated by USLII and 4643SLII-237 to manipulated hnRNP A1 level (our unpublished data). Yet hnRNP A1 belongs to a large protein family with conserved functional and structural domains; its function in EV71 replication is replaceable by hnRNP A2 [55]. Further probe of interplay between hnRNP A1 and other hnRNPs or host proteins is needed to disclose a regulatory mechanism. Differential replication ability of USLIIM and 4643SLII-237M in brain of mice also supports the hypothesis that neural-specific proteins may contribute to viral translation and virulence mentioned above. Existence of tissue-specific protein or differential binding affinities of cellular proteins to SL IIs remains uncertain.

The mouse model has been extensively used to study pathogenesis of various viruses owing to its short breeding time, low cost and easy handling. Here the mouse model recapitulated major EV71 diseases including paralysis, and led to our ferreting out Nucleotide 158 as a major determinant for virus translation and virulence in mice. We do not ambitiously imply equal contributions of the position to EV71 virulence in human infection before further testing in other animal models or human cell lines, but Nucleotide 158 may still play a key role in human EV71 circulation. Our sequence analysis revealed that all seven EV71 isolates obtained in 1986 have C at position 158, while over thirty randomly selected isolates obtained in our laboratory since 1998 have U. We also analyzed 184 EV71 sequences retrieved from Genebank to discover about 77% of these sequences possessing U (23% for C) at the same position. Given a high mutation rate of RNA virus, estimated at 1×10^−3^ to 1×10^−5^ errors per nucleotide per replication cycle [56], apparent fixation in wild virus might imply Nucleotide 158 maintaining *in vivo* fitness only in a context of other changes in virus genome. Recent study on HCV cited covariance between Nucleotide 243 in HCV 5′-UTR and amino acids in NS2/NS3 proteins. It showed HCV replication declining in response to mutation either in 5′-UTR or NS2/NS3 proteins alone, but restored in the presence of mutation in both regions [57]. Bioinformatic and functional analysis of interaction between EV71 Nucleotide 158 and other genome components may explain its significance.

Causes for contrary virulence of Isolates 237 and 4643 between humans and mice remain unclear. One direct speculation is existence of species-specific proteins in human and mice that interact differentially with viral 5′-UTR, and therefore affect viral translation, replication and virulence. Recently, scanvenger receptor B2 (SCARB2) and P-selectin glycoprotein ligand-1 (PSGL-1) were identified as cellular receptor for EV71 [37], [58]. Virulence of EV71 isolates and recombinant viruses are being tested in these receptor-transgenic mice to determine whether expression or distribution of receptor from human affect viral virulence in mice. In addition, humanized mouse model was demonstrated to recapitulate human diseases of HIV-1 and Dengue virus infection. Testing virulence in humanized mice may resolve this contradiction [59], [60], [61].

This study correlates virulence of two EV71 clinical isolates with viral 5′-UTR in a mouse infection model. Molecular determinant for this differential virulence is located in the upper SL II of EV71, 12 nucleotides in length. Point substitutions in upper SL II reveal a pivotal role of nucleotide change of C158U on EV71 virulence. Mechanism for regulation by the determinant involves altered viral translation and replication ability. Our literature search shows nucleotide 158 as both the first reported virulence determinant in EV71 5′-UTR and first position discovered from unadapted isolates, while other studies focus on adapted phenotype determinants.

## Supporting Information

Table S1 **Primer sequences used for infectious clone constructions.**
(DOC)

Table S2 **Primer sequences used for nucleotide substituted infectious clones.**
(DOC)

Table S3 **Primer sequences used for bicistronic plasmids and subgenomic replicons.**
(DOC)

Figure S1 **Correlation of 5′-UTR and EV71 virulence in mice.** Three-day-old ICR mice were i.p. inoculated with recombinant virus 237 M or N7008M at 5×10^4^ pfu/mouse. The survivals of mice were monitored for two weeks. Mock control mice were given viral medium.
(TIF)

Figure S2 **Predicted RNA secondary structure of SL II.** Secondary structures of USLII, USLII-C142U, USLII-A146G, USLII-C158U and USLII-142/159 were predicted with mfold web server with preset parameters. Alteration of secondary structure presented in USLII-C158U as arrow indicated.
(TIF)
