# Supplementary material for: A Single Nucleotide in Stem Loop II of 5′-Untranslated Region Contributes to Virulence of Enterovirus 71 in Mice
Source: PLoS One. 2011 Nov 1;6(11):e27082. doi: 10.1371/journal.pone.0027082 (PMC3206083; doi:10.1371/journal.pone.0027082)
Supplement: Table S1 — Primer sequences used for infectious clone constructions. (DOC) [file pone.0027082.s001.doc]

**Table S1.** Primer sequences used for infectious clone constructions

| **Clone** | **Primer** | **Sequence (5′ to 3′)** |
| --- | --- | --- |
| MP4-inf | 4643F* | gTAgAATTCAgCTAATACgACTCACTATAgTTAAAACAgCCTgTgggTTg |
|  | RT-50* | CCTACggCCgTTTTTTTTTTTTTTTTTTTTTTTTTTTTTTTTTTTTTTTTTTTTTTTTTTgCTATTCTggTTATAACAAATTTAC |
| 4643M | R-EV71-5UTR | gCCCATgTTTgATTgTgTTgAgg |
|  | MP4-VP4N | CAAACATgggCTCACAggTgTCC |
| 237M | 237R-EV71-5UTR | gAgCCCATgTTTAATTgTgTTgAgAgCC |
|  | 237-MP4VP4N | CACAATTAAACATgggCTCACAggTgTC |
|  | R-MP4-BbvCI* | CCACATgCCTCAgCAgATggAg |
| 237/4643M | F-SL2 | ggACTgAgTATCAATAggCTgCTCgCgCggCTgAAgg |
|  | R-SL2 | CCgCgCAAgCAgTCTATTgATACTCggTCCggggAg |
| 4643/237M | 237-191F | ggACTgAgTATCAATAgACTgCTTgCgCggTTgAAgg |
|  | 4643-202R | CCgCgCAAgCAgTCTATTgATACTCAgTCCggggAA |
| 4643CL-237M | 237-101F | CCCCTTCCCCCAgTCTgAAACATAgAAgCAATgC |
|  | 4643-100R | gTTTCAgACTgggggAAggggAgTAAAAACAggCg |
| 4643SLII-237M | 237-100R | gCTTCTAAgTTACTTCggggggAAgggATATAAAACAgg |
|  | 4643-101F | CCCTTCCCCCCgAAgTAACTTAgAAgCTgTAAATC |
|  | 4643-202R | CCgCgCAAgCAgTCTATTgATACTCAgTCCggggAA |
|  | 237-191F | ggACTgAgTATCAATAgACTgCTTgCgCggTTgAAgg |
| USLIIM | FU-SL2-42 | AACgCgCCAgTTACgTCTTgATCAAgCACTTCTgTTTCCCCg |
|  | RU-SL2-42 | AACTggCgCgTTACgCCTgCTATTgATCgTTgATTTAC |
| LSLIIM | FL-SL2-42 | GCACACCAgTCATACCATgATCAAgCACATCTgTCTCCCCggACC |
|  | RL-SL2-42 | gACTggTgTgCCACACCTgCTATTgATCAgTgATgCgCATTgC |
| USLIIM-LOOP | F-LOOP | CTgAACACACCAgTTACgTCATgATCAAgC |
|  | R-LOOP | CTggTgTgTTACgCCTgCTATTgATCAg |

*: 4643F contains cutting site for EcoRI, RT-50 contains EagI site and R-MP4-BbvCI contains BbvCI site.
