# Supplementary material for: A Single Nucleotide in Stem Loop II of 5′-Untranslated Region Contributes to Virulence of Enterovirus 71 in Mice
Source: PLoS One. 2011 Nov 1;6(11):e27082. doi: 10.1371/journal.pone.0027082 (PMC3206083; doi:10.1371/journal.pone.0027082)
Supplement: Table S2 — Primer sequences used for nucleotide substituted infectious clones. (DOC) [file pone.0027082.s002.doc]

| **Table S2.** Primer sequences used for nucleotide substituted infectious clones | | |
| --- | --- | --- |
| **Clone** | **Primer** | **Sequence (5′ to 3′)** |
| C142U | F-C142U | ATAgCAggTgTAACgCgCCAgTTACgTCTTgATCAAgC |
|  | R-C142U | gCgCgTTACACCTgCTATTgATCgTTgATTTACAgC |
| A146G | F-A146g | ggCgTAgCgCgCCAgTTACgTCTTgATCAAgCACTTCTgTTTCC |
|  | R-A146g | AACTggCgCgCTACgCCTgCTATTgATCgTTgATTTAC |
| C158U | F-C158U | CAgTTATgTCTTgATCAAgCACTTCTgTTTCCCCgg |
|  | R-C158U | CAAgACATAACTggCgCgTTACgCCTgCTATTgATCg |
| 142/159 | F-142/159 | AACACACCAgTTACgTCTTgATCAAgCACTTCTgTTTCCCCg |
|  | R-142/159 | AACTggTgTgTTACgCCTgCTATTgATCgTTgATTTAC |
| 145/156 | F-145/156 | CgTgACgCgCCAgTCACgTCTTgATCAAgCACTTCTgTTTCCCCgg |
|  | R-145/156 | CgTgACTggCgCgTCTCgCCTgCTATTgATCgTTgATTTACAgC |
| U158C | F4643-U158C | CAgTCACACCTTgATCAAgCACTTCTgTTTCCCCgg |
|  | R4643-U158C | CAAggTgTgACTggTgTgCCACACCTgCTATTgATCg |
