# Supplementary material for: A Single Nucleotide in Stem Loop II of 5′-Untranslated Region Contributes to Virulence of Enterovirus 71 in Mice
Source: PLoS One. 2011 Nov 1;6(11):e27082. doi: 10.1371/journal.pone.0027082 (PMC3206083; doi:10.1371/journal.pone.0027082)
Supplement: Table S3 — Primer sequences used for bicistronic plasmids and subgenomic replicons. (DOC) [file pone.0027082.s003.doc]

**Table S3.** Primer sequences used for bicistronic plasmids and subgenomic replicons

| **Gene** | **Primer** | **Sequence (5′ to 3′)** |
| --- | --- | --- |
| β-gal | F-NotI-gal* | gCTAgCggCCgCCATggTCgTTTTACAACgTCgTgACTgg |
|  | R-BamHI-gal* | gCggATCCTTATTATTTTTgACACCAgACC |
| Luciferase | R-XhoI-FLuc* | CCgCTCgAgTTTTTTTTTTTTTTTTTaTTaCACggCgATCTTTCCgCCC |
| Replicons | 4643F | gTAgAATTCAgCTAATACgACTCACTATAgTTAAAACAgCCTgTgggTTg |
|  | 5UTR-XmaI | TTTCCCCCCgggTgTggACACTTgTgAgCCCAT |

*: Restriction sites were shown in primer names.
