# Supplementary figures and images for: A Single Nucleotide in Stem Loop II of 5′-Untranslated Region Contributes to Virulence of Enterovirus 71 in Mice
Source: PLoS One. 2011 Nov 1;6(11):e27082. doi: 10.1371/journal.pone.0027082 (PMC3206083; doi:10.1371/journal.pone.0027082)

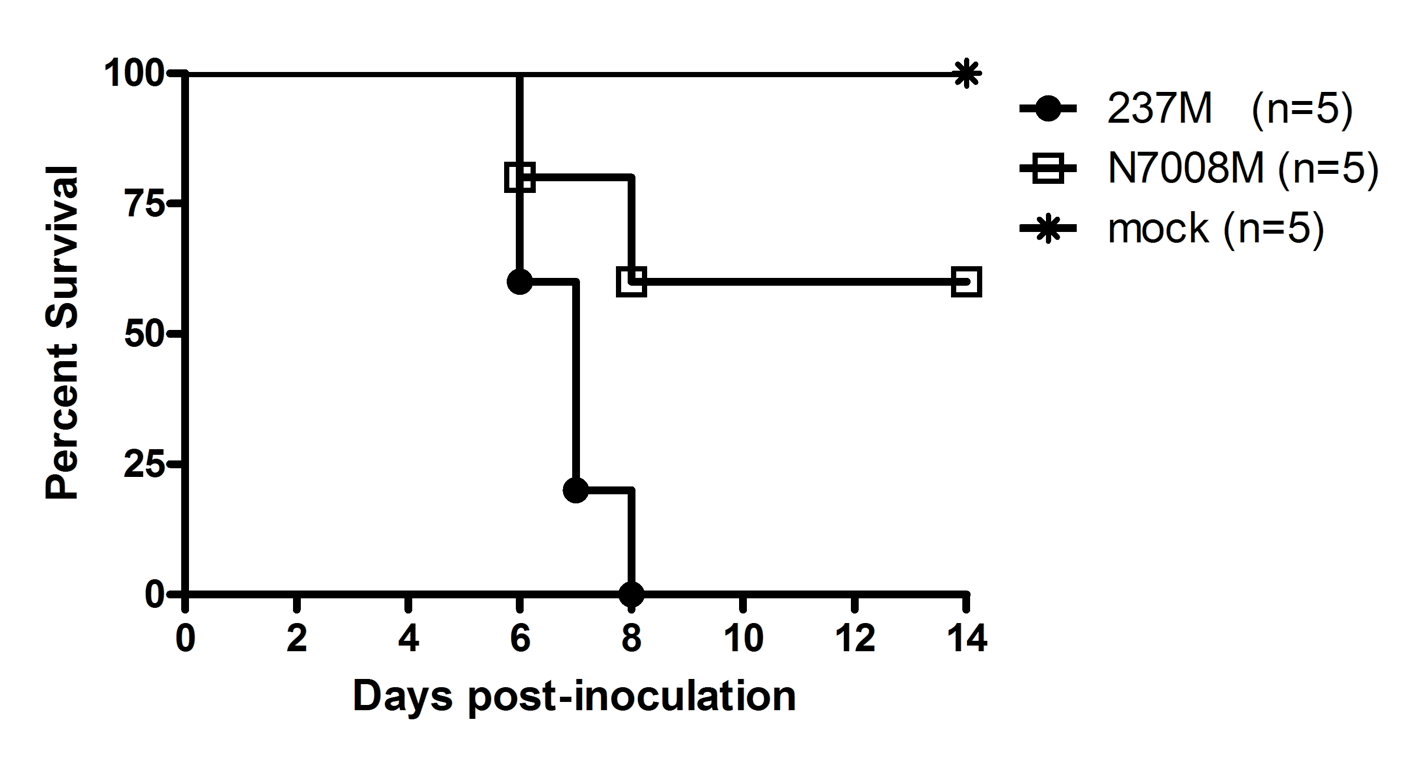

Supplement: Figure S1 — Correlation of 5′-UTR and EV71 virulence in mice. Three-day-old ICR mice were i.p. inoculated with recombinant virus 237 M or N7008M at 5×10^4^ pfu/mouse. The survivals of mice were monitored for two weeks. Mock control mice were given viral medium. (TIF) [file pone.0027082.s004.tif]

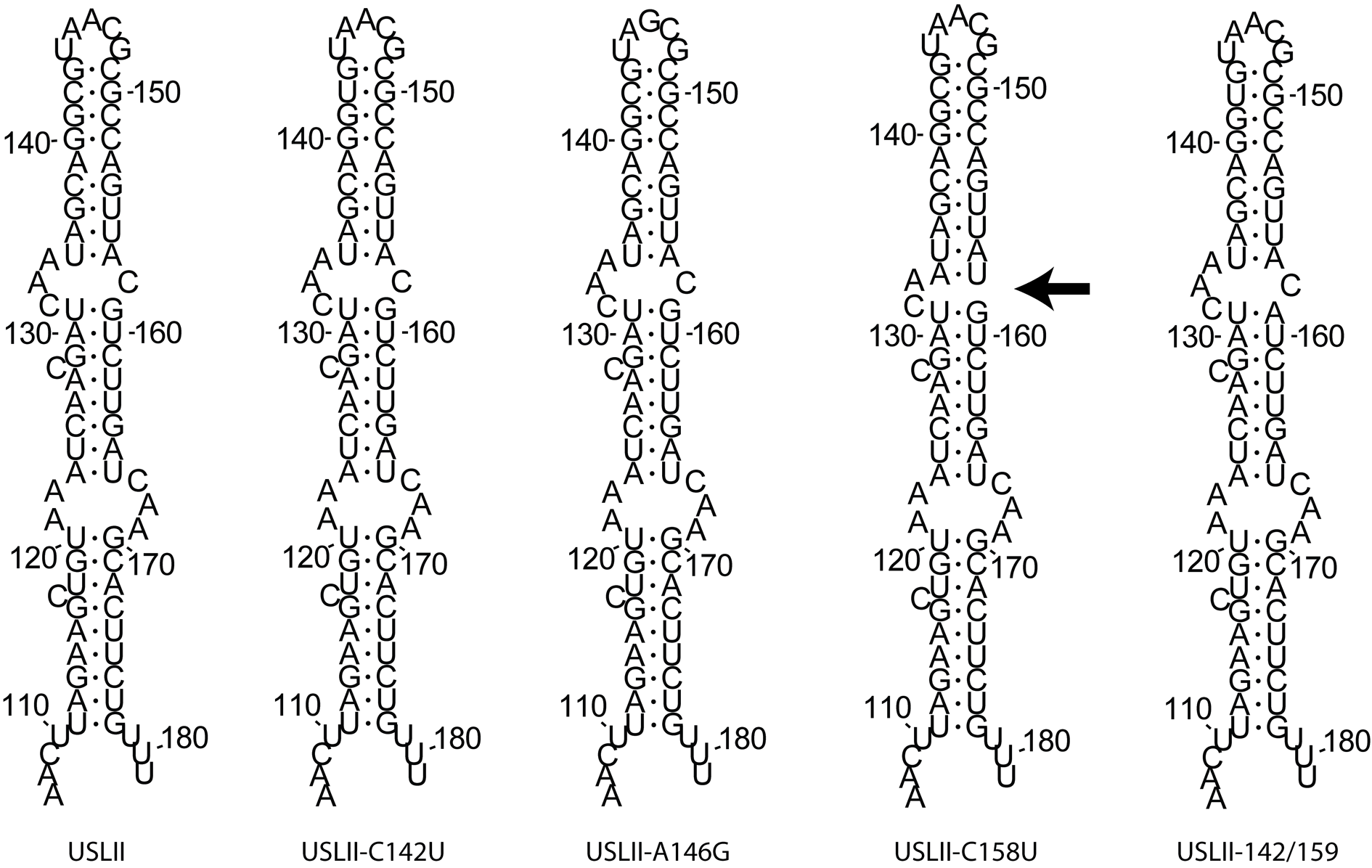

Supplement: Figure S2 — Predicted RNA secondary structure of SL II. Secondary structures of USLII, USLII-C142U, USLII-A146G, USLII-C158U and USLII-142/159 were predicted with mfold web server with preset parameters. Alteration of secondary structure presented in USLII-C158U as arrow indicated. (TIF) [file pone.0027082.s005.tif]
